# Activation of miR-9 by human papillomavirus in cervical cancer

**DOI:** 10.18632/oncotarget.2599

**Published:** 2014-10-18

**Authors:** Weijun Liu, Ge Gao, Xiaoxia Hu, Yuhui Wang, Julie K. Schwarz, Jason J. Chen, Perry W. Grigsby, Xiaowei Wang

**Affiliations:** ^1^ Department of Radiation Oncology, Washington University School of Medicine, St. Louis, Missouri; ^2^ Department of Obstetrics and Gynecology, Washington University School of Medicine, St. Louis, Missouri; ^3^ Department of Cell Biology and Physiology, Washington University School of Medicine, St. Louis, Missouri; ^4^ Alvin J. Siteman Cancer Center, Washington University School of Medicine, St. Louis, Missouri; ^5^ Department of Medicine, University of Massachusetts Medical School, Worcester, Massachusetts; ^6^ People's Hospital of Guangxi Province, Nanning, China; ^7^ Pre-clinic and Forensic Medical School, Sichuan University, Chengdu, China

**Keywords:** microRNA, miR-9, human papillomavirus, cervical cancer, expression profiling

## Abstract

Cervical cancer is the third most common cancer in women worldwide, leading to about 300,000 deaths each year. Most cervical cancers are caused by human papillomavirus (HPV) infection. However, persistent transcriptional activity of HPV oncogenes, which indicates active roles of HPV in cervical cancer maintenance and progression, has not been well characterized. Using our recently developed assays for comprehensive profiling of HPV E6/E7 transcripts, we have detected transcriptional activities of 10 high-risk HPV strains from 87 of the 101 cervical tumors included in the analysis. These HPV-positive patients had significantly better survival outcome compared with HPV-negative patients, indicating HPV transcriptional activity as a favorable prognostic marker for cervical cancer. Furthermore, we have determined microRNA (miRNA) expression changes that were correlated with tumor HPV status. Our profiling and functional analyses identified miR-9 as the most activated miRNA by HPV E6 in a p53-independent manner. Further target validation and functional studies showed that HPV-induced miR-9 activation led to significantly increased cell motility by downregulating multiple gene targets involved in cell migration. Thus, our work helps to understand the molecular mechanisms as well as identify potential therapeutic targets for cervical cancer and other HPV-induced cancers.

## INTRODUCTION

Human papillomavirus (HPV) infection can lead to a variety of human cancers [1, 2]. It has been shown that most cervical cancers are caused by HPV infection [3, 4]. Two oncogenes encoded in the HPV genome, E6 and E7 play critical roles in cervical cancer development [5]. Expression of HPV E6 leads to degradation of p53, which is a critical tumor suppressor regulating cell growth and apoptosis. Furthermore, HPV E7 binds and deactivates another important tumor suppressor, retinoblastoma protein (Rb). In most cases, expression of both E6 and E7 are required for oncogenic transformation.

In addition to deactivating tumor suppressors p53 and Rb, HPV infection leads to many cellular changes during cervical cancer development [6]. However, the impact of HPV infection on host microRNA expression has not been well characterized. MicroRNAs (miRNAs) are small non-coding RNA molecules (~23 nucleotides) that downregulate the expression of their gene targets [7]. Both computational and experimental studies indicate that thousands of human protein-coding genes are directly regulated by miRNAs. Thus, miRNAs play important regulatory roles in many physiological processes as well as a variety of disease states such as cancer [8]. Relevant to this study, altered miRNA expression profiles have been reported in cervical carcinomas as compared with normal cervix [9-15]. In addition, miRNA expression changes have been used as biomarkers for cervical cancer prognosis [16]. However, it is not clear whether these cervical cancer-related miRNA changes were directly caused by HPV infection, or simply indirect effects from cervical cancer progression in general. To specifically characterize miRNA expression changes that are related to HPV activity, we determined the expression status of HPV E6 and E7 transcripts in 101 cervical carcinomas. Transcriptional activity of HPV was then correlated to miRNA expression profiles to identify HPV-associated miRNA changes. In this way, we have identified miR-9 as the most activated miRNA by HPV E6 in cervical cancer, and showed that both the transcriptional activity of HPV E6/E7 and miR-9 were prognostic markers for cervical cancer. Further target validation and functional cell biology analyses showed that HPV-induced miR-9 activation led to significantly increased cell motility by downregulating multiple gene targets that are involved in cell migration, which may contribute to the progression of cervical cancer.

## RESULTS

### HPV transcriptional activity was a prognostic marker for cervical cancer

**Figure 1:**
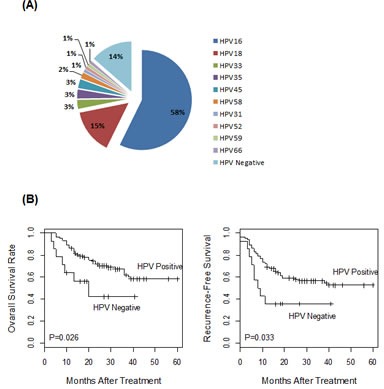
HPV expression profile in 101 cervical tumors (A) The HPV status of the cervical tumors was determined by expression profiling of the E6 and E7 transcripts from 13 high-risk HPV types. The majority of these tumors (n=59) were HPV type 16 positive. Collectively, ten HPV types were detected in 87 of the 101 tumors. (B) Prognostic significance of tumor HPV status. The 101 cancer cases were stratified by HPV status and correlated to disease outcomes, as evaluated by five-year overall survival or recurrence-free survival. Tumor HPV status was significantly associated with better patient survival.

One hundred and one cervical carcinomas were profiled for HPV expression (patient characteristics listed in Table 1). To detect and quantify HPV transcriptional activities, we have recently developed real-time PCR-based assays for expression profiling of E6 and E7 transcripts from 13 high-risk HPV types [17]. By performing these assays, HPV transcriptional activity was detected in 87 of the 101 cervical cancer cases (86.1%). Among all the HPV-positive cases, ten distinct HPV types were detected (Figure 1A). Consistent with previous studies, HPV16 and HPV18 were the most common types. In addition, eight other HPV strains were also detected, including types 31, 33, 35, 45, 52, 58, 59 and 66. For each of the 87 HPV-positive cases, both E6 and E7 transcripts were detected. For most tumors, both E6 and E7 transcripts from the same HPV strain were detected. However, double HPV infections were detected in two cases, as both E6 and E7 transcripts were detected from each of the two HPV strains, HPV16/58 and HPV16/35, respectively.

**Table 1:** Patient characteristics*

Age at diagnosis (mean ± s.d.)	51.4 ± 12.7
Tumor histology	
Squamous	89
Adenocarcinoma	10
Clear cell	2
Undifferentiated	1
FIGO stage*	
I	28
II	36
III	32
IV	5
Histological grade	
Well	4
Moderate	48
Poorly	31
Undetermined	18

* FIGO represents International Federation of Gynecology and Obstetrics.

Tumor HPV expression status was correlated to patient outcome with Cox regression analysis. As shown in Figure 1B, patients with HPV-positive tumors had significantly better survival outcome than those with HPV-negative tumors, as evaluated by both five-year overall survival and recurrence-free survival (P = 0.026 and 0.033 with the log-rank test, respectively). Thus, transcriptional activity of HPV E6 and E7 oncogenes was a prognostic marker associated with favorable outcome for cervical cancer.

### miR-9 was upregulated in HPV-positive cervical tumors

**Figure 2:**
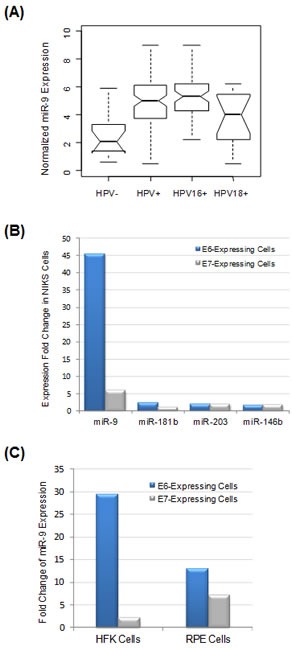
miR-9 expression was significantly correlated with HPV expression status (A) miR-9 expression profiles in four selected groups: HPV-negative tumors (n=14), all HPV-positive tumors (n=87), HPV16-positive tumors (n=57, with two double HPV infection cases excluded) and HPV18-positive tumors (n=15), respectively. (B) The impact of HPV E6 and E7 oncogenes on miRNA expression. HPV16 E6 or E7 genes were stably expressed in NIKS keratinocytes, and the impact on miRNA expression was determined by profiling analysis and comparison with negative control cells expressing vehicle vector. Among all miRNAs included in the analysis, four most upregulated ones are presented here. (C) Activation of miR-9 by HPV oncogenes in other cell types. HPV16 E6 or E7 were stably expressed in primary HFK keratinocytes and epithelium RPE cells, respectively, and the impact on miRNA expression was determined. miR-9 was the most activated miRNA in both HFK and RPE cells.

The HPV status of the 101 cervical tumors was correlated to the corresponding miRNA expression profiles, which have been determined in our previous study [16]. In this way, four HPV-related miRNAs were identified, including upregulated miR-9, miR-205 and miR-224, as well as downregulated miR-29b (Table 2). Among all miRNAs included in the analysis, miR-9 was most significantly associated with tumor HPV status (upregulated by 2.2-fold in HPV-positive tumors, false discovery rate = 0.002). miR-9 expression was further correlated to individual HPV types. As shown in Figure 2A, miR-9 was significantly upregulated in HPV-positive tumors compared with HPV-negative tumors (P = 2.7E-05 with Student's t-test). When correlated to specific HPV strains, miR-9 overexpression in HPV18-positive tumors was relatively modest and not statistically significant (P = 0.11 with Student's t-test). In contrast, miR-9 was strongly upregulated in HPV16-positive tumors compared with both HPV18-positive and HPV-negative tumors, with P = 0.007 and P = 2.2E-06 by Student's t-test, respectively.

**Table 2:** HPV-associated miRNAs in cervical cancer

miRNA Name	Fold Change	False Discovery Rate
miR-9	2.2	0.002
miR-224	1.8	0.011
miR-205	3.3	0.012
miR-29b	−0.9	0.033

The fold change values were Log2 transformed. Statistical significance was calculated with Student's t-test and the resulting p-values were further adjusted for multiple testing with the false discovery rate approach.

Interestingly, miR-9 has been identified as a biomarker for favorable prognosis in cervical cancer in our previous study [16]. Consistently, in this expanded analysis of 101 cervical cancer cases, miR-9 was also shown to be a significant prognostic marker, with P = 0.008 and 0.009 for five-year overall survival and recurrence-free survival, respectively by the log-rank test. To evaluate potential dependency of miR-9 expression on HPV transcriptional activity, the 101 patients were stratified into either HPV-positive group or HPV-negative group, and the prognostic significance of miR-9 was evaluated for each patient cohort separately. Cox regression analysis showed that miR-9 was no longer a significant marker, with P = 0.06 and 0.11 for five-year overall survival and recurrence-free survival, respectively for the HPV-positive cohort, and P = 0.54 and 0.24 for five-year overall survival and recurrence-free survival, respectively for the HPV-negative cohort. Thus, this outcome analysis indicated that the prognostic significance of miR-9 was mainly a result of its association with tumor HPV status.

### miR-9 was activated by HPV E6 oncogene

To determine HPV genes responsible for miR-9 activation, E6 and E7 genes from HPV16 were individually expressed in immortalized human foreskin keratinocytes (NIKS), which are considered as a natural host for HPV [18]. The impact of viral gene expression was determined by miRNA expression profiling and comparison with negative control cells expressing empty vehicle vector. Strikingly, miR-9 was the most activated miRNA, upregulated by 45-fold as a result of E6 expression (Figure 2B). E7 expression also led to five-fold upregulation of miR-9. In comparison, the expression changes for all other miRNAs were much smaller (< 3-fold). Thus, the *in vitro* cell profiling data were consistent with the result from cervical tumor tissue profiling, indicating that upregulation of miR-9 expression in cervical cancer was a result of HPV oncogene activity.

In addition to NIKS cell analysis, the causative role of HPV activity on miR-9 activation was also evaluated in other cell types, including primary foreskin keratinocyte HFK and retinal pigment epithelium (RPE) cells. In both cellular systems, E6 and E7 genes from HPV16 were individually expressed and the impact on miRNA expression was evaluated by profiling analysis. Interestingly, in both HFK and RPE cells, miR-9 was the most upregulated miRNA by HPV E6 oncogene (Figure 2C). Thus, miR-9 activation by HPV E6 was a general mechanism observed in multiple cell types.

### HPV-induced miR-9 activation was independent of p53 activity

**Figure 3:**
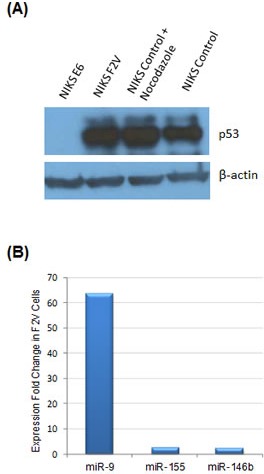
miR-9 activation by HPV E6 was independent of p53 activity (A) p53 protein expression in NIKS cells expressing HPV E6 or E6 mutant F2V by Western blot. As a positive control, nocodazole (50ng/ml) was added to the medium to upregulate p53 expression in NIKS vector control cells. p53 protein was detected in F2V-expressing cells and vector control cells, but not in E6-expressing cells. The p53 and β-actin blots were exposed for 50 and 4 seconds, respectively. (B) miR-9 was the most upregulated miRNA in F2V-expressing NIKS cells. Three most upregulated miRNAs among all miRNAs included in the analysis, as identified by expression profiling, are presented in the graph.

Deactivation of tumor suppressor p53 is a major function of HPV E6 oncogene in cervical cancer. Thus, we determined whether HPV-induced miR-9 activation is a result of p53 deactivation by E6. To this end, an E6 mutant F2V (Phe-2 to Val mutation) was studied. Our previous work indicated that F2V mutant was defective for p53 degradation but competent for immortalization of mammary epithelial cells and abrogation of cell cycle checkpoint [19, 20]. In this study, F2V mutant was expressed in NIKS keratinocytes, and the impact on miRNA expression was determined by profiling analysis. As shown in Figure 3A, p53 protein expression was suppressed by E6, but not by F2V mutant, a result consistent with our previous observations [20]. miRNA expression profile of F2V-expressing cells was determined and compared with that of E6-expressing cells. Interestingly, miR-9 was the most significantly activated miRNA by F2V, upregulated by over 60-fold as compared with the negative control vector cells (Figure 3B). Thus, miR-9 was significantly upregulated by both E6 and F2V, indicating that its activation by HPV E6 was independent of the p53 pathway.

### Identification of miR-9 targets

**Figure 4:**
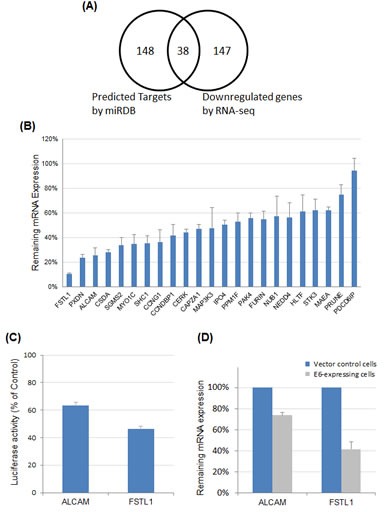
Identification and validation of miR-9 targets (A) Systematic identification of miR-9 targets by combining computational target prediction with RNA-seq expression profiling. Transcriptome-wide miRNA target prediction was performed with miRDB. In parallel, RNA-seq experiment was performed to globally identify genes downregulated by miR-9 overexpression. Among all genes expressed in HeLa cells, 38 were both predicted miR-9 targets and downregulated by miR-9 as revealed by RNA-seq. (B) Real-time RT-PCR to validate miR-9 targets. Among 23 selected target candidates, 18 were downregulated by over 40% (<60% remaining expression level). (C) Luciferase reporter assays to validate miR-9 target sites in the 3′-UTR of FSTL1 or ALCAM mRNA. The 3′-UTR sequences of FSTL1 and ALCAM were individually cloned into pmirGLO, and luciferase activities were measured after co-transfection of individual 3′-UTR constructs with miR-9 and negative control RNA, respectively. The impact of FSTL1 or ALCAM 3′-UTR on firefly luciferase activity was evaluated by comparing with the negative control and normalized by Renilla luciferase signals. (D) Downregulation of FSTL1 and ALCAM in E6-expressing NIKS cells as compared with vector control cells.

miR-9 can target a wide array of genes in a manner dependent on the cellular context [21]. To determine potential functional relevance of miR-9 activation in cervical cancer, genes targeted by miR-9 were determined by combined computational and experimental analyses. First, putative gene targets of miR-9 were computationally predicted using our previously developed algorithm, with prediction data retrieved from miRDB [22, 23]. The superior performance of miRDB over other common algorithms has recently been demonstrated in an independent comparative analysis [24]. Among all 6,487 genes expressed in cervical cancer HeLa cells, 186 were predicted to be miR-9 targets by miRDB (Figure 4A). Then, high-throughput RNA sequencing (RNA-seq) analysis was performed to globally validate these predicted gene targets in HeLa cells. Overexpression of miR-9 had a significant impact on the transcriptome of HeLa cells. Altogether, 185 genes were downregulated by at least 50%, including 38 genes that were predicted to be miR-9 targets by miRDB (Figure 4A, complete gene list presented in Supplementary Table 1). The enrichment of computationally predicted miR-9 targets among all downregulated genes (20.5%) was much higher than the transcriptome background (2.9%, P < 10^−22^ with the hypergeometric test). Thus, these 38 genes were likely to be directly targeted by miR-9.

Real-time RT-PCR experiments were performed to validate the RNA-seq results. Twenty-three potential cancer-related miR-9 targets, as identified by both RNA-seq and computational target prediction, were selected for further validation. Among them, 18 were downregulated by over 40% as validated by real-time RT-PCR (Figure 4B). Two qPCR-validated miR-9 targets, FSTL1 and ALCAM were particularly interesting, as both genes were among the most suppressed targets and have been reported to regulate cell mobility [25, 26]. Thus, FSTL1 and ALCAM were selected for further functional characterization.

Luciferase reporter assays were performed to confirm direct targeting of FSTL1 and ALCAM by miR-9. To this end, the 3′-UTR sequences of FSTL1 and ALCAM were individually cloned into pmirGLO, which is a dual-luciferase miRNA target expression vector. The luciferase activity was measured after co-transfection of the constructed expression vectors with miR-9 or negative control RNA in 293T cells. For both FSTL1 and ALCAM constructs, overexpression of miR-9 significantly suppressed the luciferase activity as compared with the negative control RNA (Figure 4C). Thus, miR-9 was able to regulate the 3′-UTR of FSTL1 or ALCAM mRNA, leading to suppression of target protein expression. Given that miR-9 was activated by HPV E6, we hypothesized that FSTL1 and ALCAM expression should be downregulated in the presence of HPV E6. To this end, the expression levels of FSTL1 and ALCAM were determined by real-time RT-PCR in HPV16 E6-expressing NIKS cells. As shown in Figure 4D, both FSTL1 and ALCAM mRNA expression levels were significantly decreased in HPV16 E6-expressing NIKS cells as compared with vector control cells. Combined together, these results indicated that FSTL1 and ALCAM were direct targets of miR-9.

### miR-9 and HPV E6 were positive regulators of NIKS cell migration

**Figure 5:**
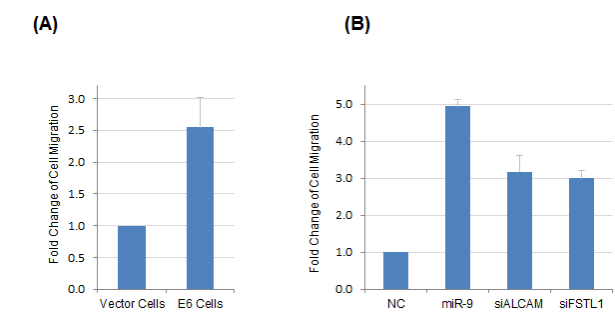
HPV E6 and miR-9 were both positive regulators of NIKS cell motility (A) HPV16 E6 expression led to increased mobility of NIKS cells as compared with control cells expressing vehicle vector. (B) Transient overexpression of miR-9 or knockdown of miR-9 targets (FSTL1 or ALCAM) by siRNA led to increased mobility of NIKS cells as compared with the negative control RNA transfection.

miR-9 has been implicated in regulation of cancer metastasis; however, it could exert opposing roles (either promoting or suppressing metastasis) depending on cancer type or cellular context [21]. To characterize the functional relevance of miR-9 activation by HPV, we evaluated the impacts of miR-9 and HPV E6 on regulating the motility of NIKS cells with transwell migration assays. To this end, expression of E6 resulted in significantly increased motility of NIKS cells by 2.5-fold as compared with control cells expressing the vehicle vector (Figure 5A). We hypothesized that the effect on cell migration by E6 was likely through activation of miR-9, as miR-9 expression was significantly upregulated in E6-expressing cells (Figure 2). To test potential direct impact of miR-9 on cell motility, miR-9 was overexpressed by transient transfection in NIKS cells that had no E6 expression. As a result, the mobility of miR-9 expressing cells was significantly increased by 4.9-fold compared with that of negative control cells (Figure 5B). Furthermore, we also determined the roles of two validated miR-9 targets, FSTL1 and ALCAM in regulating cell migration. RNAi knockdown experiment was performed to suppress the expression of FSTL1 and ALCAM in NIKS cells, respectively. The remaining mRNA levels of FSTL1 and ALCAM were 8% and 15% after siRNA knockdown, respectively as determined by real-time RT-PCR. As shown in Figure 5B, knockdown of ALCAM or FSTL1 led to increased mobility of NIKS cells (3.2-fold and 3.0-fold, respectively), which mimicked the functions of miR-9 and HPV E6 for promoting NIKS cell migration.

## DISCUSSION

### HPV transcriptional activity in cervical cancer

Although HPV genomic DNA is detected in almost all cervical cancer cases [3, 4], persistent transcriptional activity of HPV oncogenes has not been well characterized. Specifically, the presence of E6 and E7 transcripts indicates active functional roles of HPV in cervical cancer maintenance and progression. To this end, we have recently developed real-time RT-PCR assays to profile E6 and E7 from thirteen high-risk HPV types, which represents one of the most comprehensive methods to date for transcriptional profiling of HPV oncogenes [17]. With these assays, we have detected transcriptional activities of ten HPV strains from 86% of all cervical tumors included in our analysis. Interestingly, these HPV-positive patients had significantly better survival outcome compared with HPV-negative patients. Thus, HPV transcriptional activity was a favorable prognostic marker for cervical cancer. We have previously shown that HPV transcriptional activity was detected in 81% of oropharyngeal cancers and was predictive of favorable survival outcome [17]. Thus, for both types of cancer, HPV activity serves as a biomarker for favorable prognosis. This is consistent with recent observations that HPV-positive oropharyngeal tumors harbor less gene mutations and are more responsive to chemoradiation therapy compared with HPV-negative oropharyngeal tumors [27, 28]. Currently, there are multiple ongoing clinical trials to evaluate modified treatment plans for oropharyngeal cancer patients based on tumor HPV status, with the goal of improving therapeutic effectiveness. In comparison, the HPV status of cervical cancer has not been well utilized for guiding treatment decisions. In this study, we showed that a small, but significant fraction (14%) of cervical cancers were HPV negative and with poor survival outcome. These patients could potentially benefit from modified therapies as they responded poorly to standard chemoradiation treatment.

### miR-9 activation by HPV in cervical cancer

miR-9 exerts diverse roles in many types of tissue. In particular, miR-9 plays a prominent role in neurogenesis by regulating targets involved in proliferation, migration and differentiation of neural progenitor cells [21]. Relevant to this work, recent studies show that miR-9 is functionally involved in regulating cancer metastasis, either promoting or suppressing metastasis depending on the tissue context [29-32]. As to cervical cancer, inconsistent results have been reported regarding potential change of miR-9 expression compared with normal cervix tissues. One study [14] showed increased miR-9 expression in tumors while a few other studies reported no significant change (reviewed in [33]). The discrepancy is likely a result of varying proportions of HPV-infected tumor or normal control tissues included in those studies, as the HPV status of the tissues had not been determined. By age 50, at least 80% women in the US will have been infected with HPV (http://www.cdc.gov). Thus, a large proportion of tumor as well as normal cervix tissues are infected by HPV. Our work indicated that miR-9 was upregulated by HPV in both cancer tissues and non-cancer normal cells. Thus, activation of miR-9 is a result of HPV infection preceding the development of cancer. In this case, no miR-9 expression change would be observed if both tumor and normal tissues were HPV positive.

Our study showed that miR-9 was specifically activated by HPV in a p53-independent manner. However, the mechanisms of miR-9 activation by HPV are yet to be discovered. Interestingly, Wilting *et al*. showed that the miR-9 locus is associated with a frequent abnormal chromosomal gain in cervical cancer [14]. Thus, miR-9 activation in cervical cancer could be a result of elevated chromosomal instability induced by HPV infection. This hypothesis is consistent with the extensive roles of HPV played in inducing genomic instability in carcinogenesis (reviewed in [34]). However, extensive mechanistic studies would be needed to elucidate how miR-9 is activated by HPV, which is beyond the scope of the current study.

Similar to cervical cancer, we have recently reported upregulated miR-9 expression associated with HPV activity in oropharyngeal cancer [35], an observation confirmed by an independent study [36]. Thus, activation of miR-9 by HPV is a general phenomenon that is not only confined to cervical cancer. Our current work identified multiple genes that were targeted by miR-9. Both activation of miR-9 and suppression of its targets resulted in increased mobility of HPV hosting cells, which could eventually lead to metastatic cancer. Thus, our work helps to understand the molecular mechanisms as well as identify novel therapeutic targets for HPV-induced cancers including cervical cancer, oropharyngeal cancer, and potentially other types of cancer as well.

## MATERIALS AND METHODS

### Patient tumor samples

All 101 cervical cancer patients included in this study were treated at the Washington University School of Medicine in St. Louis, with patient characteristics summarized in Table 1. This study was approved by the Human Research Protection Office at the Washington University. All these archived tumor tissues have been described in detail and analyzed previously for miRNA expression studies [16]. In brief, tumor tissues were collected prior to radiotherapy and processed by the Tissue Procurement Core Facility at Washington University. Then, total RNA was isolated from the archived tumors as described in detail previously [16].

### Expression profiling of HPV and miRNAs

HPV expression profiling of the tumor samples was performed using PCR-based assays as described in our recent study [17]. In brief, these assays were designed to detect and quantify E6 and E7 transcripts from thirteen high-risk HPV types (16, 18, 31, 33, 35, 39, 45, 52, 56, 58, 59, 66, and 68). All oligonucleotide primers in the assays were purchased from Sigma-Aldrich. Reverse transcription (RT) reaction was performed with High Capacity cDNA Reverse Transcription Kit (Life Technologies). Real-time PCR was performed to quantitate the cDNA with Power SYBR Green PCR Master Mix (Life Technologies) and HPV type-specific primers. Each E6 and E7 assay from all 13 HPV types was performed separately in a 384-well PCR plate, with PCR running protocol described previously [17]. In addition, both GAPDH and β-actin expression levels were used as the internal reference control for real-time PCR data normalization.

miRNA expression profiling was performed using a PCR-based method, with detailed experimental protocol described in our previous study [37]. In brief, the RT reactions were done with the High Capacity cDNA Reverse Transcription Kit (Life Technologies). Each RT reaction included total RNA as the template and a pool of miRNA-specific RT primers. Real-time PCR was performed individually for each of 96 cancer-related miRNAs with Power SYBR Green PCR Master Mix (Life Technologies). These cancer-related miRNAs were selected based on mining of public literature [37]. Raw PCR data were normalized using a quantile-based scaling method as described previously [37].

### Survival analysis

Statistical analysis was performed with the R package (http://www.r-project.org/). Univariate Cox proportional hazards regression analysis was done to evaluate the prognostic significance of HPV or miR-9 expression in cervical tumors, and the p-values were calculated with the log-rank test. Five-year overall survival and recurrence-free survival were used as the end points to represent disease outcome. Overall survival was defined as the time interval between treatment start date and the date of death from any cause; recurrence-free survival was defined as the time interval between treatment start date and the date of death or first failure.

### Cell culture and transfection

HeLa cells were purchased from American Type Culture Collection (ATCC). Primary human foreskin keratinocytes (PHK) were prepared from neonatal foreskins obtained from the University of Massachusetts Memorial Hospital as described [38]. Immortalized human foreskin keratinocyte cells (NIKS) with stable expression of HPV E6, E6 mutant F2V or E7 were established using the pBabe retroviral system as described in our previous study [39]. Both PHK and NIKS cells were maintained on mitomycin C–treated J23T3 feeder cells in F-medium as described [39]. RPE1 is a human telomerase reverse transcriptase–expressing human retinal pigment epithelium cell line obtained from Clonetech Laboratories. RPE1 cells were cultured in 1:1 DME and Ham's F12 media (Sigma-Aldrich) as described [19]. miRNA mimics, siRNAs and negative control RNA (a random sequence) were purchased from Sigma-Aldrich. Cells were transfected with 60 nM of either miRNA or siRNA using Lipofectamine 2000 (Life Technologies) according to the manufacturer's recommended protocol.

### Western blot

Cells were lysed with RIPA buffer (Thermo Scientific). Then, the lysate was centrifuged and protein supernatant was loaded to SDS–PAGE. Protein bands on the gel were transferred to Immun-Blot PVDF membrane (Bio-Rad). The membrane was first blocked with 5% milk in TBS buffer and then incubated with primary anti-p53 mAb (BD Biosciences) or anti-β-Actin mAb (Sigma-Aldrich). After washing with TBS buffer, the blot was incubated with secondary anti-mouse IgG mAb conjugated with horseradish peroxidase (Thermo Scientific), and bound antibodies were visualized by HRP substrate (Millipore) and detected on X-ray films.

### RNA-seq

High-throughput RNA sequencing (RNA-seq) was performed as described in our previous study [40]. In brief, total RNA was extracted from the cells with the mirVana kit (Life Technologies). Ribosomal RNA (rRNA) was removed from total RNA with the RiboMinus kit (Life Technologies) and custom rRNA oligonucleotide probes. Then, the rRNA-depleted RNA samples were used to construct sequencing libraries with the NEBNext mRNA Library Prep kit (New England BioLabs). Amplified cDNA libraries were loaded into HiSeq 2000 (Illumina) for sequencing at the Genome Technology Access Center at the Washington University. Raw sequence reads were preprocessed with a custom bioinformatics pipeline for clustering before mapping to the RefSeq human transcriptome with Bowtie [41]. Mapped sequence reads were then normalized using the RPKM method (reads per kb per million), and compared across samples to evaluate changes in transcript abundance.

### Real-time RT-PCR for target validation

Reverse transcription was performed with the High Capacity RT kit (Life Technologies). All PCR primer sequences were retrieved from PrimerBank [42]. Real-time PCR was performed to validate the downregulation of candidate miRNA targets as identified by RNA-seq, using the standard PrimerBank PCR protocol as described [43]. GAPDH and β-actin were included as internal controls for expression data normalization. Potential target expression changes were determined with the 2ΔΔCt method by comparison with the negative control PCR.

### Luciferase reporter assay

The 3′-UTRs of human FSTL1 and ALCAM were amplified by PCR and cloned into pmirGLO vector (Promega). For luciferase reporter assays, each construct was co-transfected with miRNA mimics or negative control RNA (60 nM) into 293T cells in a 96-well plate with Lipofectamine 2000 (Life Technologies). Twenty-four hours post transfection, luminescent signals from firefly luciferase were determined with a Luminoskan Ascent luminometer (Thermo Fisher Scientific) and normalized by luminescent signals from Renilla luciferase.

### Transwell migration assay

Migration assays were performed using 8.0 μM polycarbonate transwell inserts (Corning) according to manufacturer's instructions. Briefly, the bottom wells were filled with 600 μL medium containing 10% bovine serum. A total of 1×10^5^ cells in 100 μl serum-free medium were plated in the upper inserts and allowed to migrate for 24 hrs. Non-migrated cells were removed with a cotton swab, while cells that had migrated were fixed and stained with Hema-3 (Fisher Scientific) for counting. The fold change of cell migration was calculated by comparing the cells expressing miR-9 and HPV E6 with the cells expressing negative control RNA and empty vehicle vector, respectively.

## SUPPLEMENTARY TABLE


